# Supplementary material for: Enhanced Photocatalytic Performance and Mechanism of Au@CaTiO3 Composites with Au Nanoparticles Assembled on CaTiO3 Nanocuboids
Source: Micromachines (Basel). 2019 Apr 17;10(4):254. doi: 10.3390/mi10040254 (PMC6523114; doi:10.3390/mi10040254)
Supplement: Supplementary file 1 [file micromachines-10-00254-s001.pdf]

Supplementary Materials: Enhanced Photocatalytic Performance and Mechanism of Au@$CaTiO_3$ Composites with Au Nanoparticles Assembled on $CaTiO_3$ Nanocuboids

Yuxiang Yan, Hua Yang, Zao Yi, Ruishan Li and Xiangxian Wang

Photoluminescence (PL) spectroscopy characterization of $CaTiO_3$ and 4.3%Au@$CaTiO_3$ was performed on a RF-6000 fluorescence spectrophotometer (Shimadzu, Kyoto, Japan) at $\lambda_{excitation}$ = 375 nm. As shown in Figure S1, an obvious PL emission peak at 555.4 nm, which arises due to the electron/hole pair recombination, is observed for both $CaTiO_3$ and 4.3%Au@$CaTiO_3$. However, the 4.3%Au@$CaTiO_3$ composite manifests a relatively weak PL emission peak, indicating a decreased electron-hole recombination in the composite. The efficient separation of electron/hole pairs can be explained as the result of the electron transfer from the conduction band (CB) of $CaTiO_3$ NCs to Au NPs.

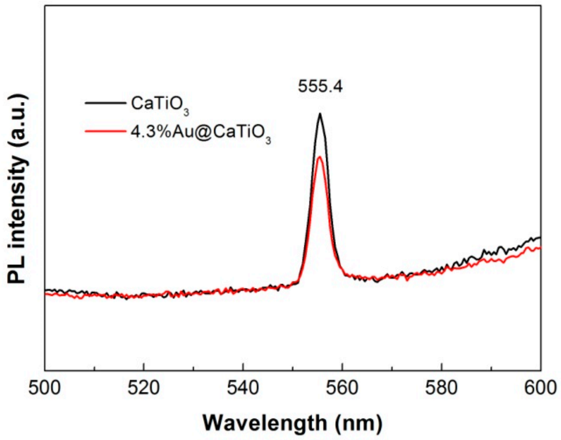

Figure S1. PL spectra of $CaTiO_3$ and 4.3%Au@$CaTiO_3$ measured at an excitation wavelength of 375 nm.

A CST 350 electrochemical workstation (Wuhan Corrtest Instruments Co. Ltd., Wuhan, China) equipped with a three-electrode cell configuration was used to study the electrochemical impedance spectroscopy (EIS) and photocurrent response of the samples. The used electrolyte was 0.1 M Na_2SO_4 aqueous solution. The used light source was a 200 W xenon lamp emitting simulated sunlight. A 0.2 V bias voltage was used during the transient photocurrent measurement. The sinusoidal voltage pulse was used for the EIS measurement (amplitude: 5 mV; frequency range: 10^{-2}–10^5 Hz).

Based on the photocurrent response and EIS analyses, We compared the separation/transfer behavior of photoexcited carriers between $CaTiO_3$ and 4.3%Au@$CaTiO_3$. Figure S2a shows the transient photocurrent-time curves of $CaTiO_3$ and 4.3%Au@$CaTiO_3$ under intermittent irradiation of simulated sunlight. An obvious photocurrent response behavior is observed for both the samples, and more importantly, the 4.3%Au@$CaTiO_3$ composite exhibits a higher photocurrent density than bare $CaTiO_3$ on the irradiation. As seem from Figure S2b, the EIS spectra (Nyquist plots) of $CaTiO_3$ and 4.3%Au@$CaTiO_3$ display a typical semicircle shape. The observed smaller semicircle diameter for the 4.3%Au@$CaTiO_3$ composite implies that it has a relatively smaller charge-transfer resistance. From the photocurrent response and EIS analyses, it is confirmed that the 4.3%Au@$CaTiO_3$ composite manifests a more efficient e^--h^+ separation and faster interface charge transfer than bare $CaTiO_3$.

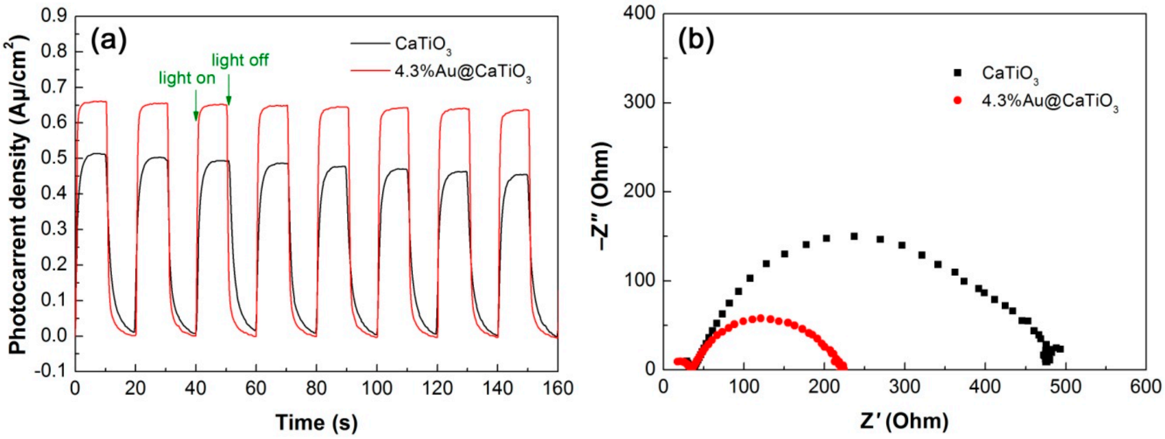

Figure S2. (**a**) Transient photocurrent response curves and (**b**) EIS spectra of $CaTiO_3$ and 4.3%Au@$CaTiO_3$.

Hydroxyl (•OH), superoxide (•O_2^-) and h^+ are generally considered to be the main active species in most of photocatalytic systems. To elucidate their role in the RhB degradation over 4.3%Au@$CaTiO_3$, reactive species trapping experiments were carried out by using ethanol as the scavenger of •OH, benzoquinone (BQ) as the scavenger of •O_2^- and ammonium oxalate (AO) as the scavenger of h^+. An amount of ethanol (5 mL), BQ (0.1 mmol) and AO (0.1 mmol) were separately added in the reaction solution (100 mL of 5 mg·L^{-1} RhB solution + 0.1 g of 4.3%Au@$CaTiO_3$). The adsorption and photocatalytic degradation experiments were performed under the procedure same to that without adding scavengers. Simulated sunlight was used the light source. As illustrated in Figure S3, the addition of BQ to the reaction solution only has a minor suppression on the photocatalytic degradation of RhB, implying that •O_2^- plays a relatively small role in the photocatalytic reactions. A great suppression on the RhB degradation is observed on the addition of ethanol or BQ, indicative of a strong dependence of the dye degradation on •OH and h^+. The main role of the photogenerated h^+ is to react with OH^- or H_2O to produce •OH. It is suggested that •OH is the dominant reactive species reacting with the dye and causing its degradation.

Recycling photocatalytic experiment was carried out to evaluate the reusability of the 4.3%Au@$CaTiO_3$ composite toward the degradation of RhB under simulated sunlight irradiation. After the completion of each photocatalytic cycle, the photocatalyst was collected and recovered by washing with deionized water and drying at 60 °C for 5 h. The recovered photocatalyst was added in 100 mL of fresh RhB solution and then irradiated for the next photocatalytic cycle. As seen from Figure S4, the 4.3%Au@$CaTiO_3$ composite still shows a high photocatalytic removal of RhB at the 4th photocatalytic cycle. After 120 min of photocatalysis, the degradation percentage of RhB reaches 96.4%, and only 3.2% is lost compared to the dye degradation at the 1st photocatalytic cycle (99.6%). This is indicative of a good recycling stability of the Au@$CaTiO_3$ composite photocatalyst.

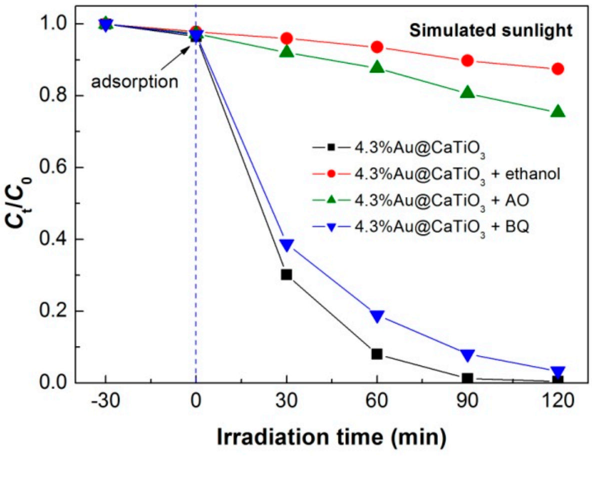

Figure S3. Effect of ethanol, BQ and AO on the degradation of RhB over 4.3%Au@$CaTiO_3$ under simulated sunlight irradiation.

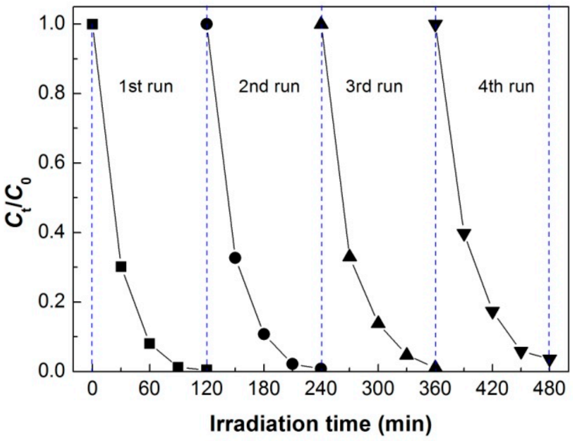

Figure S4. Reusability of 4.3%Au@$CaTiO_3$ for the RhB degradation under simulated sunlight irradiation.
